# Extract of *Allium Chinense* G. Don, a Medicinal Plant, Ameliorates Myocardial Ischemia–Reperfusion Injury by Inhibiting Platelet Activation

**DOI:** 10.3390/cimb47070503

**Published:** 2025-07-01

**Authors:** Siyuan Liu, Huaxiang Wang, Min Wang, Zhihui Wang, Na Li, Jianyong Si, Jingxue Ye

**Affiliations:** 1 Institute of Medicinal Plant Development, Peking Union Medical College and Chinese Academy of Medical Sciences, Beijing 100193, China; 2 State Key Laboratory for Quality Ensurance and Sustainable Use of Dao-di Herbs, Beijing 100193, China; 3 Key Laboratory of New Drug Discovery Based on Classic Chinese Medicine Prescription, Chinese Academy of Medical Sciences, Beijing 100193, China; 4 Beijing Key Laboratory of Innovative Drug Discovery of Traditional Chinese Medicine (Natural Medicine) and Translational Medicine, Beijing 100193, China

**Keywords:** *Allium chinense* G. Don, myocardial ischemia–reperfusion injury, platelet, aerobic glycolysis, transcriptome sequencing, molecular docking

## Abstract

*Allium chinense* G. Don is valued for its edible and medicinal qualities. It has been reported that *Allium chinense* has the potential to inhibit platelet activation, but its mechanism of action is unknown, which needs to be further explored. This study investigates the anti-myocardial ischemia–reperfusion (I/R) injury potential of *Allium chinense* from the perspective of platelet activation, focusing on its chemical composition and underlying mechanisms of action. A combination of transcriptome sequencing, molecular docking, and experimental validation was employed in our study. The antiplatelet active fraction MT-95ET of *Allium chinense* was screened by the ADP-induced platelet aggregation model in vitro. In vivo experiments demonstrated that MT-95ET can reduce the myocardial injury of I/R rats and inhibit I/R-induced platelet activation, adhesion, and aggregation. UHPLC-Q-Orbitrap-MS/MS was used to identify 13 compounds from MT-95ET. Transcriptome sequencing and molecular docking identified aerobic glycolysis key checkpoints PDK1 and PKM2 as key targets, with Sarsasapogenin and Hecogenin exhibiting strong binding affinities to these proteins. Western blot analysis further validated that MT-95ET downregulated PKM2 and PDK1, indicating a possible mechanism for its antiplatelet effects and anti-myocardial I/R injury.

## 1. Introduction

*Allium chinense* G. Don is widely used in medicinal and food applications. *Allium chinense* has been repeatedly used in a variety of traditional Chinese formulas for the treatment of coronary heart disease, such as Gualou Xiebai Baijiu decoction, Gualou Xiebai Banxia decoction, and Zhishi Xiebai Guizhi Decoction, and has been shown to have a significant therapeutic effect on myocardial ischemia–reperfusion (I/R) injury [1,2,3]. This indicates that *Allium chinense* has shown significant medicinal value in the treatment of myocardial I/R injury. However, the lack of research on the mechanism of action of *Allium chinense* indicates a need for further exploration in this area.

Myocardial I/R injury is defined as increased myocardial tissue injury following thrombolysis, percutaneous coronary intervention, and angioplasty for the treatment of myocardial ischemia [4,5,6]. Research findings indicate that the cellular and molecular mechanisms underlying myocardial I/R injury are intricate, with platelet activation being a pivotal factor in this process [7,8]. There is mounting evidence to suggest that activated platelets play a direct role in the pathogenesis of myocardial infarction and reperfusion injury by forming microthrombi, interacting with white blood cells, and releasing chemokines and inflammatory factors [9]. Antiplatelet therapy has emerged as a widely adopted preventive strategy that can improve clinical outcomes in cardiovascular disease [10].

According to *Pharmacopoeia of People’s Republic of China*, *Allium chinense* has similar pharmacological effects to *Allium macrostemon* Bunge, and they are both original plants used in traditional Chinese medicine (TCM) “Xiebai”. Research has reported the inhibitory effect of saponins from *Allium macrostemon* on platelet aggregation in vitro [11,12]. Therefore, we speculate that *Allium chinense* may have the same anti-platelet effect as *Allium macrostemon*.

In this study, we planned to use ethanol extraction to obtain the extract of *Allium chinense*, screen the active components of *Allium chinense* by the ADP-induced in vitro platelet aggregation model, establish an I/R rat model for experiments in vivo, and elucidate the effect and molecular mechanism of *Allium chinense* extract on myocardial I/R injury from the perspective of platelet activation through the combination of transcriptome sequencing, molecular docking, and experimental validation.

## 2. Materials and Methods

### 2.1. Extraction and Preparation

Fresh bulbs (60 kg) of *Allium chinense* were collected in Ganzhou City, Jiangxi Province, in September 2023 and identified by Professor Yulin Lin from the Institute of Medicinal Plant Development. The samples (ACGD202309JX) have been deposited in the Herbarium of medicinal plants, Research Center for Medicinal Plant Identification, Institute of Medicinal Plant Development. “Gualou Xiebai Baijiu Decoction” and “Gualou Xiebai Banxia Decoction” are classical traditional Chinese medicine compounds applying *Allium chinense* to treat coronary heart disease, both boiled with liquor. We use ethanol for extraction in this study. The samples were extracted under reflux with a 7-fold volume of 95% ethanol for 2 h and a 5-fold volume of 95% ethanol for 1 h sequentially. The ethanol extract was combined and concentrated under reduced pressure. The residue was mixed with kieselguhr and then extracted with organic solvents of different polarity to obtain petroleum ether fraction (PE-95ET), dichloromethane fraction (DCM-95ET), ethyl acetate fraction (EA-95ET), and methanol fraction (MT-95ET).

### 2.2. UHPLC-Q-Orbitrap-MS/MS Analysis

The active fraction MT-95ET was dissolved in methanol with a concentration of 10 mg/mL and filtered by a 0.22 μm pore-size filter membrane. The compounds of MT-95ET were qualitatively analyzed by Ultimate 3000 Ultra-High-performance Liquid Chromatograph (Dionex, Sunnyvale, CA, USA) with Thermo Q Exactive Plus High-resolution mass spectrometry (Thermo Fisher Scientific, Waltham, MA, USA).

The chromatography was performed on Waters ACQUITY UPLC HSS T3 C18 column (2.1 mm × 100 mm, 1.8 μm; Waters Corporation, Boston, MA, USA) at a column temperature of 35 °C. The mobile phase consisted of acetonitrile with 0.1% formic acid (A) and water with 0.1% formic acid (B). The following gradient elution program was used: 0–10 min, 100% B; 10–20 min, 100–70% B; 10–25 min, 70–60% B; 25–30 min, 60–50% B; 30–40 min, 50–30% B; 40–45 min, 30–0% B; 45–60 min, 0% B; 60–60.1 min, 0–100% B; 60.1–70 min, 100% B. The flow rate was set to 0.2 mL/min, and the injection volume was 2 μL.

A heated electrospray ionization source (HESI) was used for mass spectrometry. Sheath gas flow was 40 arb, auxiliary gas flow was 15 arb, capillary temperature was 320 °C, auxiliary gas heater temperature was 350 °C, positive spray voltage was 3.2 kV, and negative spray voltage was 3.0 kV. The resolution of MS was 70,000 and the resolution of MS/MS was 17,500. The scanning mode was full, and the positive and negative ion modes were detected simultaneously. The scanning range of positive ion spectra recorded by mass spectrometry was *m*/*z* 100–1500 Da.

### 2.3. Animals and Treatment

Eighty male SD rats weighing between 200 and 250 g were purchased from Beijing Vital River Laboratory Animal Technology Co., Ltd. (Beijing, China). All rats had free access to standard chow and water and were kept in a controlled environment with regulated temperature and humidity on a 12 h light/dark cycle. The experimental procedures were approved by the Laboratory Animal Ethics Committee of the Institute of Medicinal Plant Development, Peking Union Medical College (No. SLXD-20240416012) and adhered to the Guide for the Care and Use of Laboratory Animals (NIH Publication, 8th Edition, 2011). Because the model has a certain mortality rate, to ensure that each set of samples is greater than or equal to six, the 90 rats were randomly divided into six groups: the Sham group, the ischemia/reperfusion (I/R) group, the I/R + MT-95ET (40 mg/kg/day) group, the I/R + MT-95ET (80 mg/kg/day) group, the I/R + MT-95ET (160 mg/kg/day) group, and the I/R + Aspirin (50 mg/kg/day) group. Animals were administered once a day after I/R surgery (ligation of the left anterior descending coronary artery). The dosage for rats is calculated by converting the clinical dosage for humans. The MT-95ET and Aspirin were dissolved in normal saline. The experimental unit is a single animal.

### 2.4. Platelet Aggregation Rate

Blood was taken from the abdominal aorta of anesthetized SD rats, centrifuged at 200× *g* for 15 min, and the platelet-rich plasma (PRP) was separated. The platelet-poor plasma (PPP) was separated at 2000× *g* for 15 min. ADP was used to induce the aggregation of platelets, and the platelet aggregation rate was detected by the Semi-automatic platelet aggregator (TLKX, Beijing, China).

### 2.5. Histology

To measure infarct size, heart samples of rats were harvested and quickly frozen, then sectioned into slices of 4–5 μm thickness and incubated with a 2% triphenyl tetrazolium chloride (TTC) solution at 37 °C for 15 min. This process helped distinguish the area at risk (AAR), which appeared white, and its size was measured using Fiji (like ImageJ) (Java 1.5) software. To assess myocardial cell status and inflammatory cell infiltration, hearts were harvested and fixed in 10% neutral formalin. After dehydration, they were embedded in paraffin and processed into 5 μm slices. These slices were then stained with hematoxylin–eosin (HE) according to standard protocols. To evaluate the fibrotic area, cardiac slices underwent MASSON trichrome staining, also following standard protocols. The results of HE and MASSON were observed by a digital slide image scanning and analysis system (Leica Biosystems, Wetzlar, Germany).

### 2.6. Echocardiography

The Visual Sonics Vevo 770 (Visualsonics, Toronto, ON, Canada) ultra-high-resolution small animal ultrasound imaging system was utilized to evaluate the structure and function of rat hearts. The procedure involved anesthetizing the rats, applying depilatory cream to the thoracic area, and securing the animals in a supine position on the console. A coupling agent was then used, and the ultrasound probe was placed on the left side of the chest. After adjusting the probe, the left ventricular long-axis view and M-mode curve were obtained. From this data, the ejection fraction (EF) and left ventricular short-axis shortening (FS) were calculated.

### 2.7. Serum Myocardial Enzyme

Blood was collected from the abdominal aorta of anesthetized SD rats, centrifuged at 3500 rpm for 15 min, and the serum was separated. The level of CK-MB, LDH, and CK was detected by the AU480 automatic biochemical analyzer (Beckman Coulter, Brea, CA, USA) according to the instructions provided with the kits (BIOSINO, Beijing, China).

### 2.8. Flow Cytometry

Flow cytometry was utilized to measure P-selectin expression, mitochondrial membrane potential, and Ca^2+^ concentrations. Washed rat platelets were incubated with an anti-P-selectin antibody (Biolegend, San Diego, CA, USA), along with the JC-1 loading probe (Beyotime, China) and Fluo-3 AM (Beyotime, China), following the instructions provided with the kits. The samples were detected by flow cytometry (BD Biosciences, New York, NY, USA) and analyzed using FlowJo (10.8.1) software.

### 2.9. ELISA

Blood samples were collected from the abdominal aorta of anesthetized SD rats and then centrifuged at 3500 rpm for 15 min to separate the serum. The levels of CXCL5 (Elabscience, Wuhan, China), TXA2 (Elabscience, China), IL-1α (Elabscience, China), and 5-HT (Elabscience, China) in the serum of rats were detected using a microplate reader (Infinite M1000, Tecan, Switzerland) according to the instructions provided with the ELISA kit.

### 2.10. Transcriptome Sequencing

The total RNA extracted from platelets was first tested for concentration and purity before being sequenced on the DNBSEQ platform. The sequencing data were filtered using fastp, and differential genes were screened and analyzed with STAR. The data have been uploaded to NCBI (PRJNA1236488).

### 2.11. Molecular Docking

The 2D structure of the small molecule ligand was obtained from the PubChem database (http://pubchem.ncbi.nlm.nih.gov/) (accessed on 26 March 2025), and the 2D structure was input into Chem Office (2019) software to create its 3D structure and was saved as a mol2 file. Then, the RCSB PDB database (http://www.rcsb.org/) (accessed on 26 March 2025) was used to screen the crystal structure of the protein target with high resolution as the molecular pair acceptor, and the protein was dehydrogenated and dephosphorylated using PyMOL(3.0) software and saved as a PDB file. Molecular docking was performed using AutoDock Vina 1.5.6 software to explore protein–ligand interactions. The structures of proteins as well as small molecules were processed using AutoDock to hydrogenate and dehydrate the proteins and to hydrogenate and determine the torsion force of the small molecule ligands, etc., after which the coordinates of the docking boxes were determined. The optimal conformation of the molecular simulation was finally obtained by comparing the size of the scores of the docking results. The 2D plots and 3D analytical plots of the interactions between the test compounds and key residues were visualized using PyMOL (3.0) and Discovery Studio software (2019).

### 2.12. ATP and Lactate

The level of ATP (Beyotime, Shanghai, China) and lactate (Beyotime, Shanghai, China) in platelets was detected by the Infinite M1000 microplate reader (Tecan, Männedorf, Switzerland) according to the instructions of the kits.

### 2.13. Western Blot

Total proteins were extracted and separated using SDS-PAGE (CoWin Biotech, Taizhou, China), after which they were transferred onto PVDF membranes. The membranes were then incubated with primary antibodies at 4 °C overnight. Following this, HRP-conjugated secondary antibodies were applied at room temperature for an additional 1.5 h. Finally, the membranes were visualized using the E-Blot System with an electrochemiluminescence reagent. The primary antibodies in this article were used, including PDK1 (5662T, 1:1000), P-PDK1 (3061, 1:1000), PKM2 (60268-1-Ig, 1:1000), PI3K (R381092, 1:1000), P-PI3K (12456T, 1:1000), GSK3β (12456T, 1:1000), P-GSK3β (5558T, 1:1000), β-Actin (AC006, 1:2000), and GAPDH (AC027, 1:10000).

### 2.14. Statistical Analysis

Data were collected and analyzed blindly and were presented as mean ± SEM. Statistical analyses were performed using GraphPad Prism 5.0. For the column diagrams, one-way ANOVA followed by Tukey’s post hoc test was used for multiple comparisons. Two-way ANOVA was used to compare patch-clamp and cardio ECR data. The statistical significance was set at *p* < 0.05 (two-tailed).

## 3. Results

### 3.1. Screening and Chemical Characterization by UHPLC-Q-Orbitrap-MS/MS of Anti-Platelet Active Fractions of Ethanol Extract of Allium Chinense

**Figure 1:**
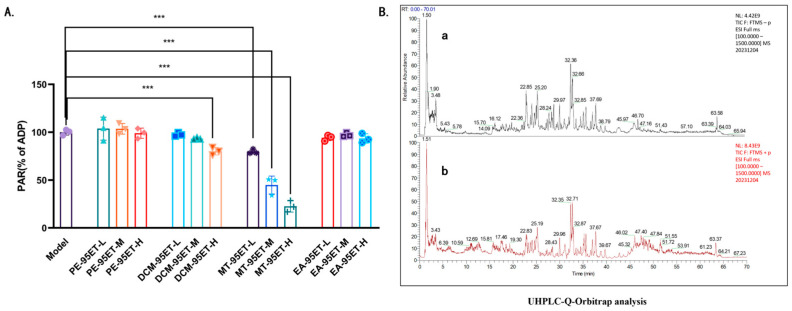
The active fraction MT-95ET. (**A**) Different polarity fractions (20 mg/mL, 40 mg/mL, and 80 mg/mL) inhibited ADP (150 μM)-induced platelet aggregation in vitro. Values are mean ± SEM. *n* = 3 individual donors per group; one-way ANOVA followed by Tukey test. *** *p* < 0.001 vs. the model group. (**B**) Total ion current chromatograms of MT-95ET in negative (**a**) and positive (**b**) ion modes.

ADP was utilized to induce platelet aggregation. Both DCM-95ET and MT-95ET can inhibit platelet aggregation in vitro. MT-95ET is more effective, but the effects of DCM-95ET were minimal (Figure 1A). Figure 1B displays the total ion current chromatograms (TIC) of MT-95ET in both positive and negative ion modes, while Table 1 provides the corresponding compound information. The Excalibur (4.3) software was utilized to analyze the chemical compounds of MT-95ET. MS^1^ and MS^2^ mass spectrometry data of MT-95ET were collected by UHPLC-MS. The relative molecular masses and fragment ions were compared with data found in the literature, leading to the identification of 13 compounds, which include 7 saponins and sapogenins.

### 3.2. MT-95ET Alleviates Myocardial Injury and Improves Cardiac Function in I/R SD Rats

**Figure 2:**
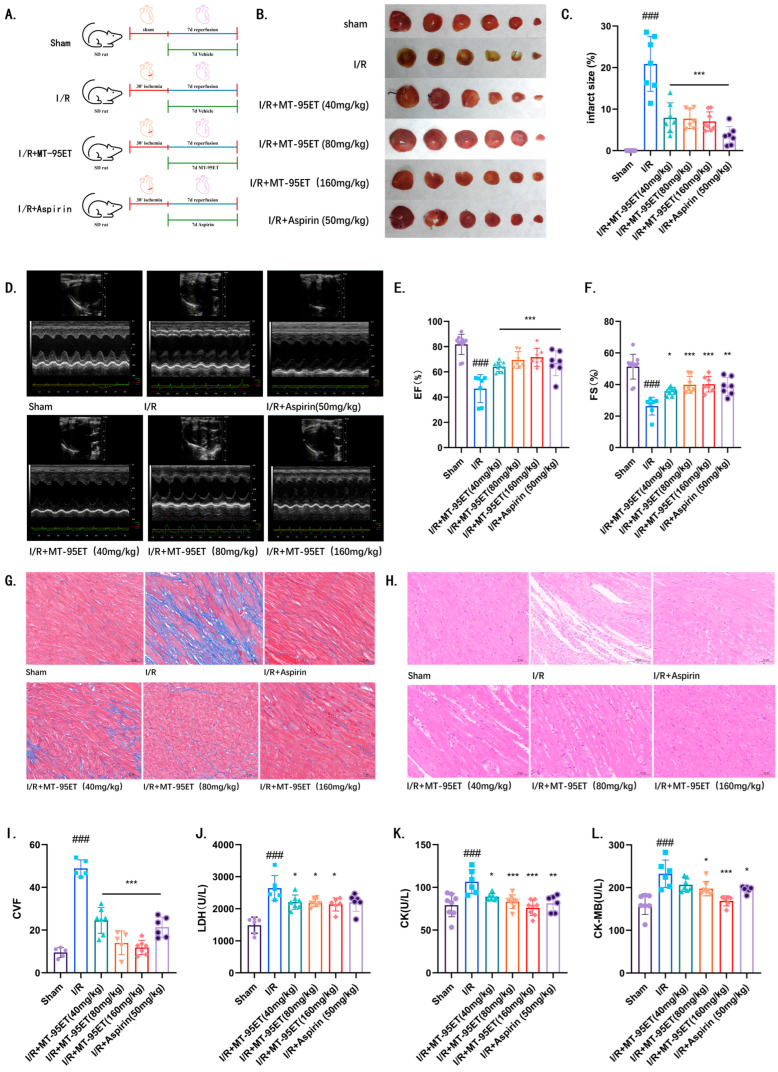
MT-95ET alleviates myocardial injury and improves cardiac function in I/R SD rats. (**A**) All rats were randomly divided into six groups: Sham group; I/R group; I/R + MT-95ET (40 mg/kg) group; I/R + MT-95ET (80 mg/kg) group; I/R + MT-95ET (160 mg/kg) group; I/R + Aspirin (50 mg/kg) group. Only Sham surgery was performed in the Sham group; the I/R + MT-95ET group was subjected to daily irrigation stomach of MT-95ET (40, 80, 160 mg/kg/d) for 7 days after the I/R operation. The I/R+ Aspirin group was subjected to a daily irrigation stomach of Aspirin (50 mg/kg/d) for 7 days after the I/R operation. (**B**) TTC staining results of cardiac cross-section. The white part represents the heart infarction, and the red part is the healthy part of the heart. (**C**) The ratio of myocardial infarction area to total area. Values are mean ± SEM. *n* = 7–8 individual donors per group; one-way ANOVA followed by Tukey test; ^###^ *p* < 0.001 vs. the Sham group; *** *p* < 0.001 vs. the I/R group. (**D**) Representative images of the cardiac functional parameters on day 7 post-myocardial I/R. (**E**) EF (%). Values are mean ± SEM. *n* = 7–11 individual donors per group; one-way ANOVA followed by Tukey test; ^###^ *p* < 0.001 vs. the Sham group; *** *p* < 0.001 vs. the I/R group. (**F**) FS (%). Values are mean ± SEM. *n* = 7–11 individual donors per group; one-way ANOVA followed by Tukey test; ^###^ *p* < 0.001 vs. the Sham group; * *p* < 0.05, ** *p* < 0.01; *** *p* < 0.001 vs. the I/R group. (**G**) Masson staining showed that collagen fibers were blue and muscle fibers were red; the scale bar is 50 µm. (**H**) HE stains of longitudinal sections of the heart; *n* = 6–7 individual donors per group; the scale bar is 50 µm. (**I**) CVF Collagenvolume fraction. Values are mean ± SEM. *n* = 5–7 individual donors per group; one-way ANOVA followed by Tukey test; ^###^ *p* < 0.001 vs. the Sham group; *** *p* < 0.001 vs. the I/R group. (**J**) LDH (U/L). Values are mean ± SEM. *n* = 6–7 individual donors per group; one-way ANOVA followed by Tukey test; ^###^ *p* < 0.001 vs. the Sham group; * *p* < 0.05 vs. the I/R group. (**K**) CK (U/L). Values are mean ± SEM. *n* = 6–7 individual donors per group; one-way ANOVA followed by Tukey test; ^###^ *p* < 0.001 vs. the Sham group; * *p* < 0.05, ** *p* < 0.01; *** *p* < 0.001 vs. the I/R group. (**L**) CK-MB (U/L). Values are mean ± SEM. *n* = 6–7 individual donors per group; one-way ANOVA followed by Tukey test; ^###^ *p* < 0.001 vs. the Sham group; * *p* < 0.05; *** *p* < 0.001 vs. the I/R group.

TTC staining revealed that the area of myocardial infarction in the I/R group was significantly increased compared to the Sham group. After 7 days of treatment with MT-95ET, the myocardial infarction area decreased obviously, showing a significant difference compared to the I/R group (Figure 2B,C). Echocardiography was used to assess the cardiac systolic function of the rats. In comparison to the Sham group, both EF and FS in the I/R group were significantly decreased, indicating abnormal cardiac systolic function. Treatment with MT-95ET resulted in a significant increase in both EF and FS (Figure 2D–F), with statistically significant differences (*p* < 0.001). This suggests that the cardiac dysfunction in the rats was alleviated to some extent. Histopathological analysis via MASSON staining demonstrated that myocardial fibrosis significantly worsened in the I/R group compared to the Sham group, while treatment with MT-95ET improved this condition (Figure 2G,I). Furthermore, HE staining results indicated that the myocardial cells in the Sham group were arranged neatly without any inflammatory infiltration. In contrast, the myocardial structure in the I/R group was disrupted, accompanied by inflammatory infiltration. However, the MT-95ET treatment group exhibited a significant reduction in myocardial injury and inflammatory cell infiltration when compared to the I/R group (Figure 2H). Additionally, the levels of creatine kinase (CK), creatine kinase isoenzyme (CK-MB), and lactic dehydrogenase (LDH) in serum were assessed in each group of rats. The I/R group displayed significantly increased levels of CK, CK-MB, and LDH compared to the Sham group. Treatment with MT-95ET, as well as with the positive control drug Aspirin, successfully reduced the levels of these enzymes (Figure 2J–L).

### 3.3. MT-95ET Plays a Role in Inhibiting I/R-Induced Platelet Activation in Vivo

**Figure 3:**
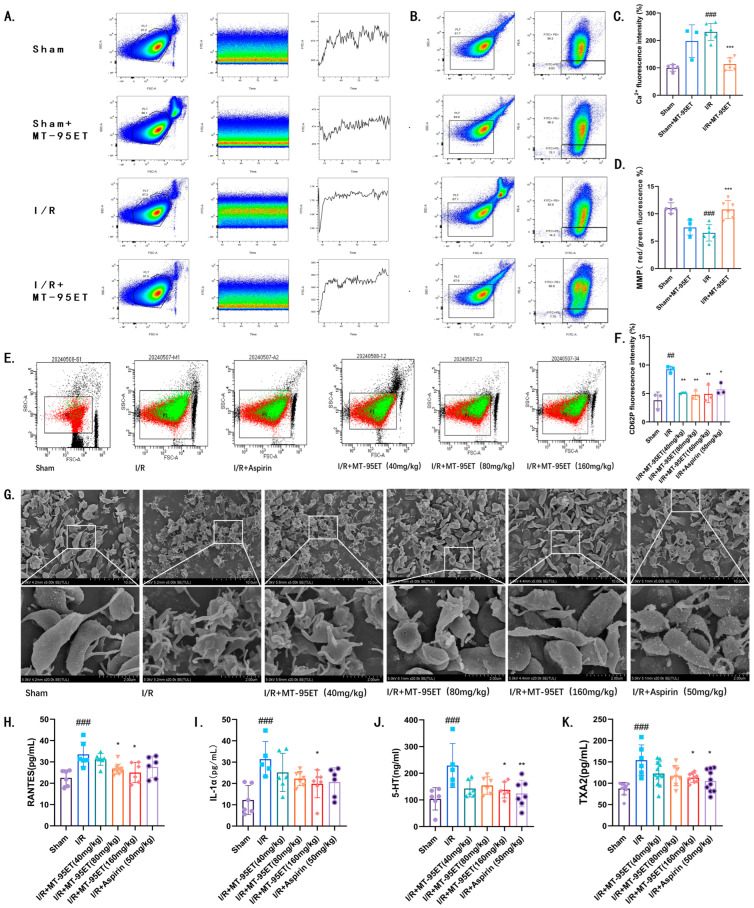
MT-95ET regulates I/R-induced platelet activation in vivo. (**A**) Platelet Ca^2+^ level was measured via Fluo-3 AM staining by flow cytometry. (**B**) Platelet mitochondrial membrane potential (MMP) was measured via JC-1 staining by flow cytometry. (**C**) Ca^2+^ fluorescence intensity (% of control). Values are mean ± SEM. *n* = 3–6 individual donors per group; one-way ANOVA followed by Tukey test; ^###^ *p* < 0.001 vs. the Sham group; *** *p*< 0.001 vs. the I/R group. (**D**) MMP (red/green fluorescence%). Values are mean ± SEM. *n* = 4–6 individual donors per group; one-way ANOVA followed by Tukey test; ^###^ *p* = 0.0003 < 0.001 vs. the Sham group; *** *p* = 0.0004 < 0.001 vs. the I/R group. (**E**) Detection of platelet CD62p expression by flow cytometry. (**F**) CD62P fluorescence intensity (%). Values are mean ± SEM. *n* = 3 individual donors per group; one-way ANOVA followed by Tukey test; ^##^ *p* < 0.01 vs. the Sham group; * *p* < 0.05, ** *p* < 0.01 vs. the I/R group. (**G**) Observation of platelet morphology by scanning electron microscope. (**H**) RANTES (pg/mL). Values are mean ± SEM. *n* = 6–10 individual donors per group; one-way ANOVA followed by Tukey test; ^###^ *p* < 0.001 vs. the Sham group; * *p* < 0.05 vs. the I/R group. (**I**) IL-1α (pg/mL). Values are mean ± SEM. *n* = 6–7 individual donors per group; one-way ANOVA followed by Tukey test; ^###^ *p* < 0.001 vs. the Sham group; * *p* < 0.05 vs. the I/R group. (**J**) 5-HT (ng/mL). Values are mean ± SEM. *n* = 6–7 individual donors per group; one-way ANOVA followed by Tukey test; ^###^ *p* < 0.001 vs. the Sham group; * *p* < 0.05; ** *p* < 0.01 vs. the I/R group. (**K**) TXA2 (pg/mL). Values are mean ± SEM. *n* = 6–12 individual donors per group; one-way ANOVA followed by Tukey test; ^###^ *p* < 0.001 vs. the Sham group; * *p* < 0.05 vs. the I/R group.

Antiplatelet therapy has been shown to improve clinical outcomes in cardiovascular disease [13]. By detecting Ca^2+^, the key messenger of platelet activation [14], we found that the concentration of platelet calcium (Ca^2+^) in I/R rats was significantly higher than that in Sham rats. Under the treatment of MT-95ET, platelet Ca^2+^ concentration decreased (Figure 3A,C). We assessed the mitochondrial membrane potential of platelets and observed a decrease in I/R rats. Depolarization of mitochondrial membrane potential is a key step in platelet microparticle formation [15]. This indicates that I/R promotes the formation of platelet microparticles, while treatment with MT-95ET appeared to inhibit this condition (Figure 3B,D). Additionally, we measured the expression of P-selectin, a marker of platelet activation, using flow cytometry. The results indicated that the expression of P-selectin in the I/R group was significantly increased. P-selectin expression of platelets in the I/R + MT-95ET group was lower than that in the I/R group (Figure 3E,F). We also observed morphological changes in platelets using a scanning electron microscope. In comparison to the control group, platelets in the model group exhibited significant deformation and produced filopodia, which was alleviated by the treatment of MT-95ET (Figure 3G).

The activation of platelets leads to the release of inflammatory factors and chemokines, which can trigger thrombin inflammation and exacerbate myocarditis, further damaging the myocardium. We measured the levels of chemokine ligand 5 (CCL5/RANTES), interleukin-1 alpha (IL-1α), 5-hydroxytryptamine (5-HT), and thromboxane A2 (TXA2) in the serum of rats using ELISA. Compared to the Sham group, the levels of these four factors were significantly increased in the serum of the I/R group, while MT-95ET treatment significantly reduced them (Figure 3H–K).

### 3.4. MT-95ET Regulates I/R-Induced Platelet Function in Vivo

**Figure 4:**
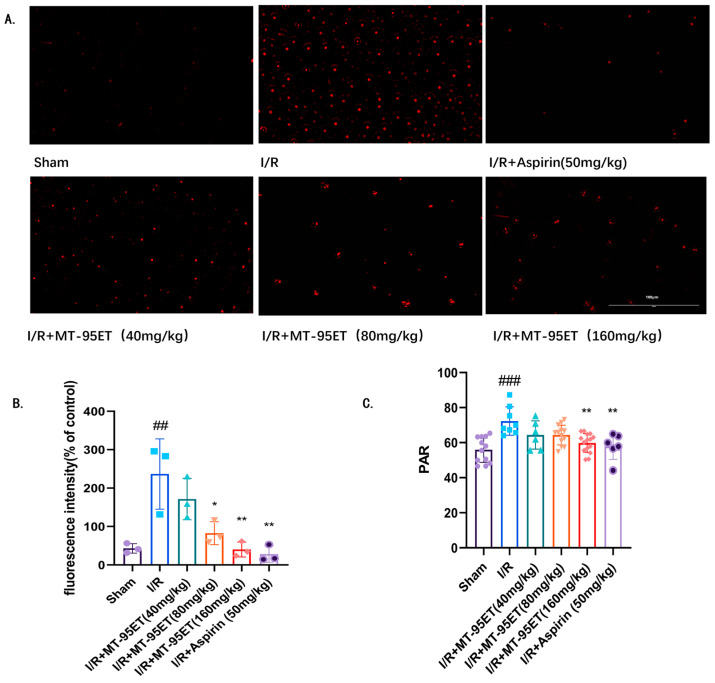
MT-95ET regulates I/R-induced platelet functional abnormality in vivo. (**A**) The adhesion function of platelets was observed by fluorescence microscopy using the ghost pen cyclic peptide staining method. (**B**) Fluorescence intensity (% of control). Values are mean ± SEM. *n* = 3 individual donors per group; one-way ANOVA followed by Tukey test; ^##^ *p* = 0.0029 < 0.01 vs. the Sham group; * *p* = 0.016 < 0.05, ** *p* = 0.0016 < 0.01 vs. the I/R group. (**C**) PRP of SD rats in each group was extracted to be induced by ADP (150 μM) to detect the rate of platelet aggregation. Values are mean ± SEM. *n* = 6–15 individual donors per group; one-way ANOVA followed by Tukey test; ^###^ *p* < 0.001 vs. the Sham group; ** *p* < 0.01 vs. the I/R group.

Platelets can exert adhesion after activation, and we used phalloidin to stain platelets to detect their adhesion function. As shown in Figure 4A,B, platelet adhesion was significantly increased in the I/R group compared with the Sham group (*p* < 0.01), which was significantly inhibited by treatment with MT-95ET and Aspirin (*p* < 0.01). It is suggested that MT-95ET inhibits I/R-induced platelet adhesion to fibrinogen. Activated platelets adhere to each other to form platelet clusters, which is known as platelet aggregation. We found that the platelet aggregation rate in I/R rats was significantly higher than in Sham rats. Conversely, the platelet aggregation rate in the I/R + MT-95ET group was considerably lower than in the I/R group, aligning with our results of in vitro experiments (Figure 1A and Figure 4C).

### 3.5. Analysis of Transcriptome Sequencing Results

**Figure 5:**
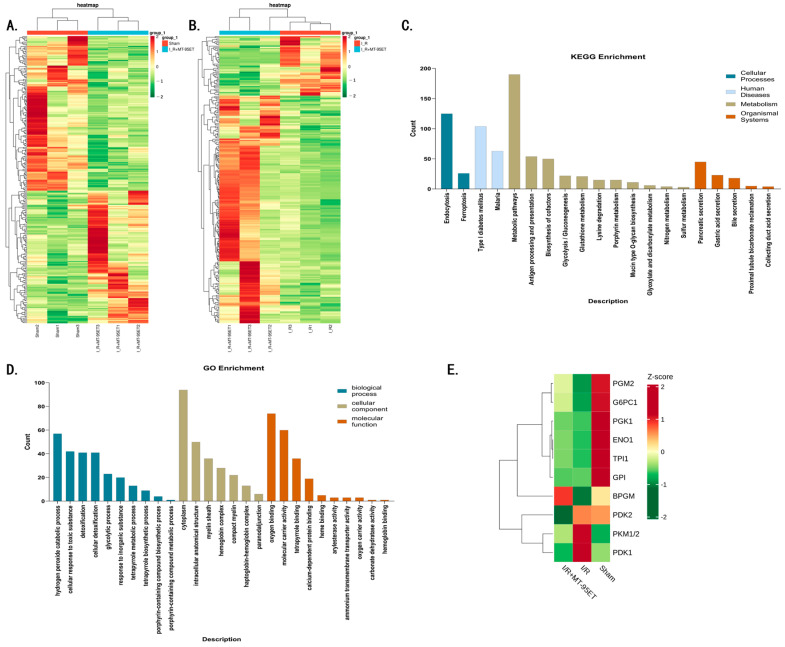
Transcriptome sequencing of platelet. (**A**) Heat map of differentially expressed genes between the Sham group and I/R group. (**B**) Heat map of differentially expressed genes between the I/R group and I/R + MT-95ET group. (**C**) KEGG enrichment analysis of MT-95ET signaling pathway on platelet activation. Enrichment analysis of differentially expressed genes pathway barplot. In the figure, the horizontal axis is the number of differentially expressed genes, the vertical axis is the KEGG pathway, and the color represents classification. (**D**) Go enrichment analysis of differential gene categories of MT-95ET on platelet activation. Barplot of GO enrichment analysis of differentially expressed genes. In the figure, the vertical axis is the number of differentially expressed genes, the horizontal axis is gene ontology, and the color represents a classification. (**E**) Z-score-based gene expression heatmap: Differential expression analysis of platelet glycolytic pathway-related genes between the I/R, I/R + MT-95ET, and Sham groups. The Z-score values are color-coded, with red representing high expression (a Z-score of 2 indicates a high degree of high expression) and green representing low expression (a Z-score of -2 indicates a high degree of low expression).

In this study, platelet transcriptome sequencing technology was used to investigate the molecular mechanism of MT-95ET against platelet activation. As shown in Figure 5A,B, there were significant differences in expressed genes between Sham and I/R groups, and between I/R and MT-95ET + I/R groups. KEGG enriched signaling pathways that were significantly different between platelets in the I/R group and the MT-95ET + I/R group, among which the glycolytic pathway was closely related to platelet activation (Figure 5C). It has been shown that the transition of platelets from the resting to the activated state is dependent on aerobic glycolysis (Warburg effect) for the short-term acquisition of large amounts of ATP [16]. As shown in Figure 5D, GO classification indicated that genes significantly differentially expressed in platelets between the I/R and MT-95ET + I/R groups also included relevant genes involved in glycolytic metabolic processes. As shown in Figure 5E, we listed significant differential genes related to the glycolytic pathway between the Sham group, the I/R group, and the MT-95ET + I/R group. We found that the expressions of PGM2, G6PC1, and BPGM were significantly lower in the I/R group than in the Sham group and were elevated under MT-95ET treatment; the expressions of PKM1/2 and PDK1 were significantly higher in the I/R group than in the Sham group and decreased under MT-95ET treatment. It is suggested that MT-95ET may act on the key genes of the glycolytic metabolic pathway, such as PGM2, G6PC1, BPGM, PKM1/2, and PDK1, to inhibit I/R-induced platelet activation.

### 3.6. Analysis of Molecular Docking Results

PKM2 and PDK1 have been reported to be key targets in the regulation of aerobic glycolytic metabolism in activated platelets [17,18]. Molecular docking simulation is a powerful tool for exploring the optimal binding modes between protein receptors and small-molecule active compounds. In this study, molecular docking was performed between the key active compounds identified through UHPLC-Q-Orbitrap-MS/MS (compound information is provided in Table 1).

**Figure 6:**
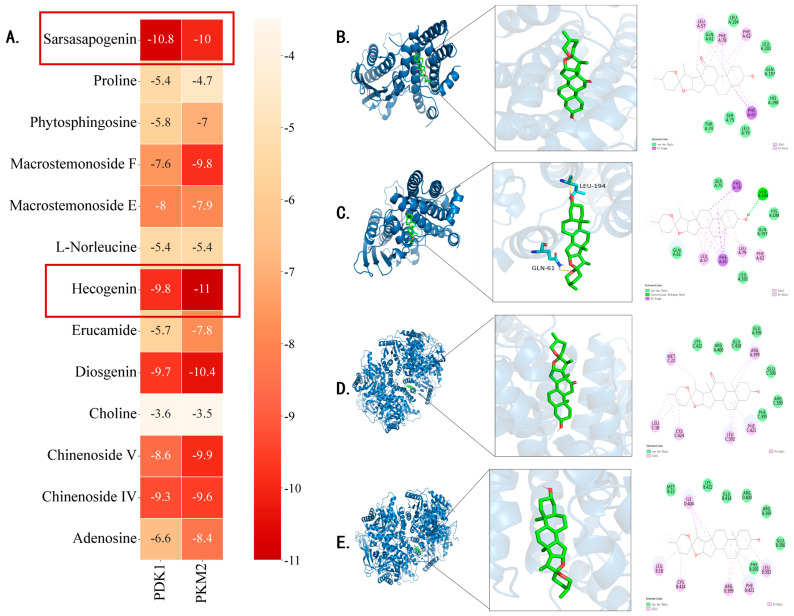
Results of molecular docking. (**A**) Heat map of protein binding energy distribution of PKM2 and PDK1 by 13 compounds. The binding energy is less than −5.0 kcal/mol, indicating good binding activity, and less than −7.0 kcal/mol, indicating strong binding activity. (**B**) PDK1_2q8g-Hecogenin, BE: −9.8 kcal/mol; (**C**) PKM2_3g2g-Hecogenins, BE: −11.0 kcal/mol; (**D**) PDK1_2q8g-Sarsasapogenin, BE: −10.8 kcal/mol; (**E**) PKM2_3g2g-Sarsasapogenin, BE: −10.0 kcal/mol.

As shown in Figure 6A, the seven compounds (Sarsasapogenin, Macrostemonoside F, Macrostemonoside E, Hecogenin, Diosgenin, Chinenoside V, Chinenoside IV) that bind well to the target protein are all saponins. Figure 6A illustrates the primary types of interactions between receptor proteins and small-molecule active compounds, including hydrogen bonds and van der Waals forces. For instance, residues GLN61, LEU194, LEU201, and GLN197 on the PDK1_2q8g receptor form van der Waals interaction force with Hecogenin (Figure 6B); the residues LYS422, ARG400, GLU418, GLU396, and ARG399 on the PKM2_3g2g receptor form van der Waals interaction force with Hecogenin (Figure 6C); the residue LEU194 on the PDK1_2q8g receptor forms a hydrogen-bonding interaction with Sarsasapogenin; the Ser75, HIS198, and GLN197 residues on the PDK1_2q8g receptor form van der Waals interaction with Sarsasapogenin (Figure 6D); residues MET22, LYS422, GLU418, ARG400, ARG399, and PHE395 on the PKM2_3g2g receptor form van der Waals forces with Sarsasapogenin (Figure 6E).

Ligands bind to specific sites on protein receptors through hydrogen bonds, van der Waals forces, hydrophobic forces, and Pi-Sigma forces interactions, which form the basis of numerous biological processes such as signal transduction, enzymatic catalysis, and hormone activity. These binding sites are typically composed of amino acid residues that provide a structurally and chemically complementary environment, ensuring high specificity and affinity for the correct ligand. The molecular docking results indicated that these compounds exhibited good docking scores with the core targets. Ligand–receptor interactions play a critical role in determining receptor functionality, as different ligands can bind to distinct receptors or different sites within the same receptor, thereby triggering diverse biological responses. For example, the bioactive compounds from Allium chinense extract may exert their anti-platelet effects by modulating key signaling pathways through strong interactions with PKM2 and PDK1 at distinct binding sites. Therefore, Sarsasapogenin and Hecogenin may be the key active components responsible for the inhibitory effects of platelet activation.

### 3.7. MT-95ET Inhibits the Aerobic Glycolysis in Activated Platelets by Regulating the Expression of PKM2 Dimer, and the Phosphorylation of PDK1, PI3K, and GSK3β

**Figure 7:**
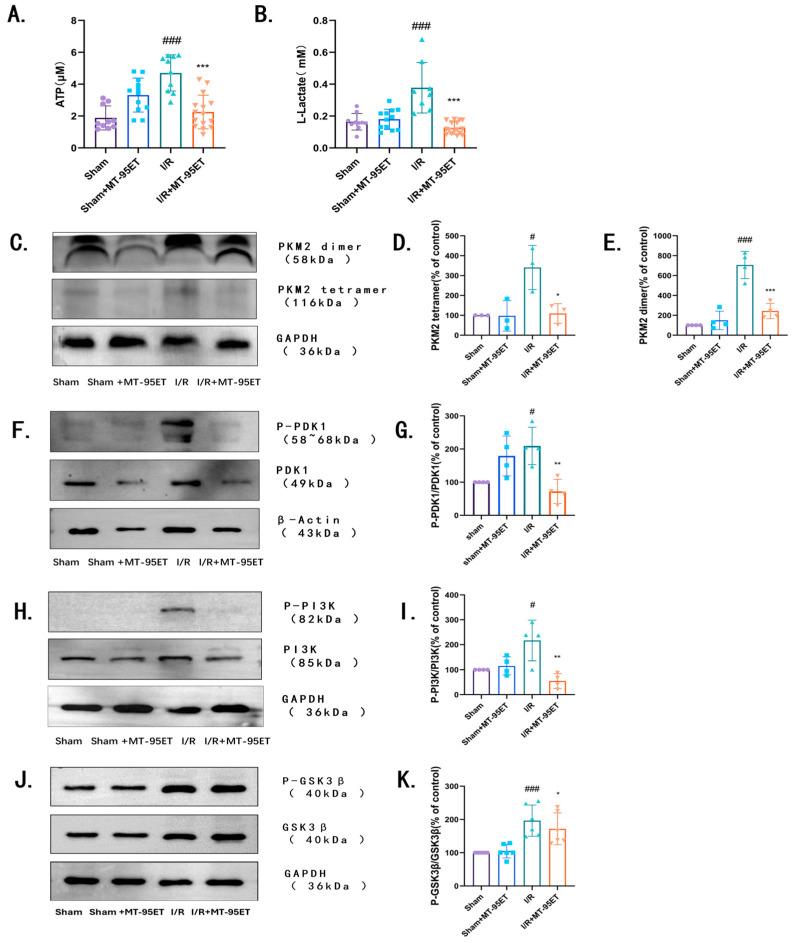
MT-95ET (160 mg/kg) inhibits the aerobic glycolysis in activated platelets by regulating the expression of PKM2 dimer, and the phosphorylation of PDK1, PI3K, and GSK3β. (**A**) ATP (μM). Values are mean ± SEM. *n* = 7–12 individual donors per group; one-way ANOVA followed by Tukey test; ^###^ *p* < 0.001 vs. the Sham group; *** *p* < 0.001 vs. the I/R group. (**B**) L-Lactate (mM). Values are mean ± SEM. *n* = 7–12 individual donors per group; one-way ANOVA followed by Tukey test; ^###^ *p* < 0.001 vs. the Sham group; *** *p* < 0.001 vs. the I/R group. (**C**–**E**) WB for PKM2 (dimer and tetramer). Values are mean ± SEM. *n* = 3 individual donors per group; one-way ANOVA followed by Tukey test; ^#^ *p* < 0.05, ^###^ *p* < 0.001 vs. the Sham group; * *p* < 0.05, *** *p* < 0.001 vs. the I/R group. (**F**,**G**) WB for PDK1 and P-PDK1. Values are mean ± SEM. *n* = 4 individual donors per group; one-way ANOVA followed by Tukey test; ^#^ *p* < 0.05 vs. the Sham group; ** *p* < 0.01 vs. the I/R group. (**H**,**I**) WB for PI3K and P-PI3K. Values are mean ± SEM. *n* = 4 individual donors per group; one-way ANOVA followed by Tukey test; ^#^ *p* < 0.05 vs. the Sham group; ** *p* < 0.01 vs. the I/R group. (**J**,**K**) WB for GSK3β and P- GSK3β. Values are mean ± SEM. *n* = 6 individual donors per group; one-way ANOVA followed by Tukey test; ^###^ *p* < 0.001 vs. the Sham group; * *p* < 0.001 vs. the I/R group.

Based on the results of transcriptome sequencing, MT-95ET may regulate platelet activation by modulating platelet aerobic glycolytic metabolism. To verify this, we extracted platelets from each group of rats to detect their cytoplasmic ATP and lactate concentrations. Our results showed that, compared to the Sham group, I/R indeed enhances the level of ATP and lactate in the platelet. However, treatment with MT-95ET significantly reduces the level of metabolites (Figure 7A,B). It is suggested that MT-95ET can inhibit platelet aerobic glycolytic metabolism.

To validate molecular docking results, we extracted platelets’ proteins and assessed the impact of MT-95ET on the target proteins PKM2 and PDK1 through Western blot experiments. Our findings revealed an increase in the expression of PKM2 dimers in the platelets of I/R rats, along with elevated phosphorylation levels of PDK1. However, treatment with MT-95ET resulted in a decrease in the expression of PKM2 dimers, as well as a reduction in the phosphorylation levels of PDK1 (Figure 7C–G). PI3K and GSK3β are the key regulatory factors in platelet exo-endosomal signaling, which can distinguish different extracellular signals and related receptors [19]. The results show that the Western blot data indicate that the phosphorylation levels of PI3K and GSK3β are upregulated in I/R-induced activated platelets, while MT-95ET can downregulate the phosphorylation of PI3K and GSK3β in I/R-induced activated platelets, thereby inhibiting platelet signaling (Figure 7J,K).

## 4. Discussion

Acute myocardial infarction is the leading cause of death and disability worldwide. Timely and effective myocardial reperfusion, achieved through decoction solution therapy or direct percutaneous coronary intervention, can lead to myocardial cell death, a phenomenon known as myocardial reperfusion injury. Currently, there is no effective treatment for this condition [20]. In myocardial I/R injury, platelets adhere to the dysfunctional endothelium and form a thrombus. They accumulate around the ischemic myocardium, obstructing microcirculation and contributing to myocardial dysfunction. Furthermore, platelets release inflammatory mediators, which block microcirculation and increase tissue inflammation, ultimately hindering the recovery of heart function [21,22]. An increasing number of patients with cardiovascular disease, particularly those with coronary artery disease, are being prescribed oral antiplatelet medications to prevent major adverse events [23,24]. *Allium chinense* is a key ingredient in Gualou Xiebai Banxia decoction and is commonly used in the clinical treatment of myocardial I/R injury, demonstrating significant therapeutic effects [1,2,3]. The pharmacological effects of *Allium macrostemon* and *Allium chinense* are similar. Existing studies have shown that the pharmacological effects of *Allium macrostemon* mainly focus on antiplatelet aggregation [25,26,27,28]. In this study, we confirmed that MT-95ET can inhibit platelet activation and alleviate myocardial I/R injury in I/R rats.

Current antiplatelet therapies, including Centorx and P2γ12 inhibitors, are effective at reducing the risk of thrombosis in patients with acute coronary syndrome. However, these treatments also significantly increase the risk of bleeding, which limits their long-term use [24]. The ideal antiplatelet drugs should selectively inhibit thrombosis while preserving the basic hemostatic mechanisms. In this context, there has been considerable interest in studying whether the metabolic pathways in targeted platelets, especially aerobic glycolysis and oxidative phosphorylation (OXPHOS), can regulate their activation to prevent thrombosis. Regardless of agonist stimulation, platelet activation is an energy-intensive process driven by ATP. It is well known that the transition of platelets from the resting state to the activated state promotes the rapid uptake of exogenous glucose and increased production of lactate. It is characterized by a transition from OXPHOS metabolism to rapid aerobic glycolysis (conversion of glucose to lactate in the presence of oxygen—Warburg effect) [17]. We found that activated platelets in the I/R group showed higher levels of lactate and ATP than platelets in the Sham group, and MT-95ET can reverse this phenomenon. It is suggested that MT-95ET can inhibit I/R-induced platelet activation by regulating platelet aerobic glycolysis metabolism.

PDK and PKM2 preferentially drive pyruvate flux for aerobic glycolysis, which are important metabolic checkpoints in aerobic glycolysis. PKM2 dimer shows low enzyme activity and acts as a metabolic switch in aerobic glycolysis. PKM2 is responsible for catalyzing the last step of glycolysis; it consumes phosphoenolpyruvate (PEP) and ADP, generates ATP, and provides pyruvate for subsequent mitochondrial oxidative phosphorylation or lactate fermentation [29]. PDK1 is the key factor between glycolysis and the Krebs cycle. When stimulated by specific agonists, PDK phosphorylates the E1α subunit of PDH, inhibiting its activity. This inhibition shifts pyruvate flux away from the Krebs cycle towards aerobic glycolysis, resulting in the production of lactate [18]. In this study, the results of our Western blot experiments showed that MT-95ET could inhibit platelet aerobic glycolysis regulators PKM2 and PDK1, thereby cutting off the energy generation process required for platelet activation and exerting an antiplatelet activation effect.

PI3K and GSK3β are the major regulators of outside-in signaling and can discriminate between diverse extracellular signals and associated receptors [19]. It is known that PDK1 and PKM2 are related to the signal transduction of platelets. PDK1 regulates αIIbβ3 integrin signaling via AKT, inhibiting GSK3β to modulate thrombin-induced platelet aggregation and clot retraction [18]. Research shows that PKM2 regulates platelet function through the PI3K-mediated AKT/GSK3 signaling pathway [16]. Our data showed that the phosphorylation levels of PI3K and GSK3β in activated platelets were upregulated, and MT-95ET downregulated the phosphorylation of PI3K and GSK3β in activated platelets, inhibited platelet signaling, and inhibited platelet activation. Suggesting that MT-95ET inhibits platelet activation by a new regulatory pathway, which coordinates many aspects of platelet function, from metabolism to cell signal transduction to platelet activation.

Our findings made up for the blank in the chemical and pharmacological research of *Allium chinense*, provided a certain experimental database for the medicinal development of *Allium chinense*, and provided a potential strategy for antiplatelet therapy of myocardial I/R injury.

There are limitations in this study. Although the detailed analysis of transcriptomic data provides evidence of a potential role for modulation of glycolysis, there is an absence of direct functional validation. To address these gaps, subsequent studies should attempt to prove causation by using gene silencing (siRNA) or pharmacologic blockers of PKM2/PDK1.

## 5. Conclusions

MT-95ET, the ethanol extract of *Allium chinense*, exhibited significant beneficial effects on myocardial injury and platelet activation induced by I/R. We conducted a comprehensive analysis of the anti-platelet effects of *Allium chinense*, utilizing a multifaceted approach that included transcriptome sequencing, molecular docking, and experimental validation to elucidate the underlying mechanisms. The UHPLC-Q-Orbitrap-MS/MS analysis identified 13 chemical constituents within the *Allium chinense* extract. Furthermore, the integration of transcriptome sequencing and molecular docking analyses revealed key targets associated with the anti-platelet effects of the *Allium chinense* extract, including PDK1 and PKM2, which present novel aerobic glycolytic metabolism targets for inhibiting platelet activation. By elucidating the anti-platelet potential of *Allium chinense* extract and its mechanisms of action, our study provides a theoretical foundation for its application as a functional food with antiplatelet and anti-myocardial I/R injury.

## Figures and Tables

**Table 1:** The information of identified compounds in MT-95ET was detected by UHPLC-Q-Orbitrap-MS/MS.

No.	RT [min]	CAS Number	Name	Formula	Reference Ion	*m*/*z*	Annot. DeltaMass [ppm]	Fragment Ions
1	1.42	62-49-7	Choline	C_5_H_13_NO	[M + H]^+1^	104.1071	0.55	104.1070, 60.0811, 58.0655
2	1.51	147-85-3	Proline	C_5_H_9_NO_2_	[M + H]^+1^	116.0707	1.21	116.0706, 70.0653
3	4.29	327-57-1	L-Norleucine	C_6_H_13_NO_2_	[M + H]^+1^	132.1020	0.76	86.0965
4	10.01	58-61-7	Adenosine	C_10_H_13_N_5_O_4_	[M − H]^+1^	268.1041	0.48	136.0619, 119.0352, 57.0338
5	27.28	151140-39-5	Macrostemonoside E	C_57_H_94_O_28_	[M − H]^−1^	1225.5859	0.46	1063.5342, 901.4816, 739.4290, 577.3757, 161.0457
6	31.29	187144-80-5	Chinenoside IV	C_50_H_80_O_23_	[M − H]^−1^	1047.5024	1.13	885.4520, 723.3990, 161.0457
7	32.67	170739-22-7	Chinenoside V	C_45_H_72_O_19_	[M − H]^−1^	915.4602	1.36	753.4063, 591.3551, 161.0457
8	33.47	512-04-9	Diosgenin	C_27_H_42_O_3_	[M + H]^+1^	415.3207	0.15	415.3205, 271.2057, 253.1951
9	35.29	554-62-1	Phytosphingosine	C_18_H_39_NO_3_	[M + H]^+1^	318.3003	0.12	318.3002, 300.2895, 282.2790, 60.0448, 56.0499
10	36.52	151215-11-1	Macrostemonoside F	C_45_H_74_O_18_	[M − H]^−1^	901.4816	2.15	739.4288, 577.3756, 161.0457
11	37.78	126-19-2	Sarsasapogenin	C_27_H_44_O_3_	[M + H]^+1^	417.3362	−0.34	417.3398, 274.2244, 273.2212, 255.2106, 97.0648
12	43.59	467-55-0	Hecogenin	C_27_H_42_O_4_	[M + H]^+1^	431.3155	−0.24	413.3049, 395.2947, 299.2371, 281.2268
13	51.49	112-84-5	Erucamide	C_22_H_43_NO	[M + H]^+1^	338.3417	−0.21	338.3410, 321.3156, 303.3047, 149.1326

## Data Availability

The data from transcriptome sequencing have been uploaded to NCBI (PRJNA1236488). The other raw data have been uploaded to Mendeley Data (https://data.mendeley.com/preview/h4hzxcx6s4?a=ca331866-5b58-4c1f-bc88-0b309c929093, accessed on 25 March 2025).

## Abbreviations

The following abbreviations are used in this manuscript:

I/R	ischemia–reperfusion
ADP	adenosine diphosphate
CAD	coronary artery disease
CK	creatine kinase
CK-MB	creatine kinase isoenzyme
CCL5/RANTES	chemokine ligand 5
EF%	left ventricular ejection fraction
ELISA	enzyme-linked immunosorbent assay
FS%	left ventricular short-axis shortening rate
GO	gene ontology
GSK3β	glycogen synthase kinase-3β
HE	Hematoxylin–eosin
IL-1α	interleukin-1α
KEGG	Kyoto encyclopedia of genes and genomes
LDH	lactic dehydrogenase
MS	mass spectrometry
MS^1^	primary mass spectrum
MS^2^	secondary mass spectrum
OXPHOS	oxidative phosphorylation
PDH	pyruvate dehydrogenase
PDK1	inositol 3-phosphate dependent protein kinase 1
PEP	phosphoenolpyruvate
PI3K	phosphatidylinositol 3-kinase
PKM2	pyruvate kinase isozyme typeM2
PPCI	percutaneous coronary intervention
SD	Sprague Dawley
TCM	traditional Chinese medicine
TIC	total ion current chromatograms
TXA2	thromboxane A2
UHPLC-Q-Orbitrap	ultra-high-performance liquid chromatography–quadrupole orbitrap
UPLC	ultra-high performance liquid chromatography
WB	Western Blot

## References

[1] Weng J., Xiong S., Jiang S., Zheng F., Lin C., Zhan L. (2023). Myocardial protective effect and mechanism of pretreatment with Gualou Xiebai Banxia Decoction on rats with myocardial ischemia-reperfusion injury model. Pract. J. Card. Cereb. Pneumal Vasc. Dis..

[2] Li X., Zhang H., Cui H., Sui Y., Li Y., Tang D. (2020). Study on the effects of Gualou Xiebai Banxia Decoction on autophagy and PINK1/Parkin pathway in rats with myocardial ischemia-reperfusion injury. Chin. J. Basic Med. Tradit. Chin. Med..

[3] Shi Y., Yang G. (2016). Mechanism of PI3K/Akt pathway in myocardium of rats with ischemia-reperfusion protected by pretreatment with Gualou Xiebai Banxia Decoction. Chin. J. Basic Med. Tradit. Chin. Med..

[4] Eltzschig H.K., Eckle T. (2011). Ischemia and reperfusion—From mechanism to translation. Nat. Med..

[5] Roth G.A., Mensah G.A., Johnson C.O., Addolorato G., Ammirati E., Baddour L.M., Barengo N.C., Beaton A.Z., Benjamin E.J., Benziger C.P. (2020). Global Burden of Cardiovascular Diseases and Risk Factors, 1990–2019: Update from the GBD 2019 Study. J. Am. Coll. Cardiol..

[6] Dauerman H.L., Ibanez B. (2021). The Edge of Time in Acute Myocardial Infarction. J. Am. Coll. Cardiol..

[7] Turer A.T., Hill J.A. (2010). Pathogenesis of myocardial ischemia-reperfusion injury and rationale for therapy. Am. J. Cardiol..

[8] Ziegler M., Hohmann J.D., Searle A.K., Abraham M.K., Nandurkar H.H., Wang X., Peter K. (2018). A single-chain antibody-CD39 fusion protein targeting activated platelets protects from cardiac ischaemia/reperfusion injury. Eur. Heart J..

[9] Wang L., Liu Y., Tian R., Zuo W., Qian H., Wang L., Yang X., Liu Z., Zhang S. (2023). What do we know about platelets in myocardial ischemia-reperfusion injury and why is it important?. Thromb. Res..

[10] Ziegler M., Wang X., Peter K. (2019). Platelets in cardiac ischaemia/reperfusion injury: A promising therapeutic target. Cardiovasc. Res..

[11] Ou W.C., Chen H.F., Zhong Y., Liu B.R., Liu S.M., Chen K.J. (2012). Inhibition of platelet activation and aggregation by furostanol saponins isolated from the bulbs of Allium macrostemon Bunge. Am. J. Med. Sci..

[12] Ling S., Jin L., Li S., Zhang F., Xu Q., Liu M., Chen X., Liu X., Gu J., Liu S. (2020). Allium macrostemon Saponin Inhibits Activation of Platelet via the CD40 Signaling Pathway. Front. Pharmacol..

[13] Ridker P.M., Cook N.R., Lee I.M., Gordon D., Gaziano J.M., Manson J.E., Hennekens C.H., Buring J.E. (2005). A randomized trial of low-dose aspirin in the primary prevention of cardiovascular disease in women. N. Engl. J. Med..

[14] Berridge M.J., Bootman M.D., Roderick H.L. (2003). Calcium signalling: Dynamics, homeostasis and remodelling. Nat. Rev. Mol. Cell Biol..

[15] Morelli A., Donati A., Ertmer C., Rehberg S., Kampmeier T., Orecchioni A., Di Russo A., D’Egidio A., Landoni G., Lombrano M.R. (2011). Effects of vasopressinergic receptor agonists on sublingual microcirculation in norepinephrine-dependent septic shock. Crit. Care.

[16] Nayak M.K., Ghatge M., Flora G.D., Dhanesha N., Jain M., Markan K.R., Potthoff M.J., Lentz S.R., Chauhan A.K. (2021). The metabolic enzyme pyruvate kinase M2 regulates platelet function and arterial thrombosis. Blood.

[17] Flora G.D., Nayak M.K., Ghatge M., Chauhan A.K. (2023). Metabolic targeting of platelets to combat thrombosis: Dawn of a new paradigm?. Cardiovasc. Res..

[18] Chen X., Zhang Y., Wang Y., Li D., Zhang L., Wang K., Luo X., Yang Z., Wu Y., Liu J. (2013). PDK1 regulates platelet activation and arterial thrombosis. Blood.

[19] Gawaz M., Geisler T., Borst O. (2023). Current concepts and novel targets for antiplatelet therapy. Nat. Rev. Cardiol..

[20] Hausenloy D.J., Yellon D.M. (2013). Myocardial ischemia-reperfusion injury: A neglected therapeutic target. J. Clin. Investig..

[21] McFadyen J., Peter K., Fitridge R. (2020). Platelets in the Pathogenesis of Vascular Disease and Their Role as a Therapeutic Target. Mechanisms of Vascular Disease: A Textbook for Vascular Specialists.

[22] Wittstein I.S. (2010). Depression, anxiety, and platelet reactivity in patients with coronary heart disease. Eur. Heart J..

[23] Kuliczkowski W., Witkowski A., Polonski L., Watala C., Filipiak K., Budaj A., Golanski J., Sitkiewicz D., Pregowski J., Gorski J. (2009). Interindividual variability in the response to oral antiplatelet drugs: A position paper of the Working Group on antiplatelet drugs resistance appointed by the Section of Cardiovascular Interventions of the Polish Cardiac Society, endorsed by the Working Group on Thrombosis of the European Society of Cardiology. Eur. Heart J..

[24] Korte W., Cattaneo M., Chassot P.G., Eichinger S., von Heymann C., Hofmann N., Rickli H., Spannagl M., Ziegler B., Verheugt F. (2011). Peri-operative management of antiplatelet therapy in patients with coronary artery disease: Joint position paper by members of the working group on Perioperative Haemostasis of the Society on Thrombosis and Haemostasis Research (GTH), the working group on Perioperative Coagulation of the Austrian Society for Anesthesiology, Resuscitation and Intensive Care (ÖGARI) and the Working Group Thrombosis of the European Society for Cardiology (ESC). Thromb. Haemost..

[25] Chen H., Ou W., Wang G., Wang N., Zhang L., Yao X. (2010). New steroidal glycosides isolated as CDL inhibitors of activated platelets. Molecules.

[26] Feng H., Wang Z., Wang C., Zhu X., Liu Z., Liu H., Guo M., Hou Q., Chu Z. (2019). Effect of Furostanol Saponins from Allium Macrostemon Bunge Bulbs on Platelet Aggregation Rate and PI3K/Akt Pathway in the Rat Model of Coronary Heart Disease. Evid.-Based Complement. Altern. Med. eCAM.

[27] Xu J., Zhang M., Lin X., Wang Y., He X. (2020). A steroidal saponin isolated from Allium chinense simultaneously induces apoptosis and autophagy by modulating the PI3K/Akt/mTOR signaling pathway in human gastric adenocarcinoma. Steroids.

[28] Xu J., Wang Y., Wang Y., Wang Z., He X. (2021). A-24, a steroidal saponin from Allium chinense, induced apoptosis, autophagy and migration inhibition in p53 wild-type and p53-deficient gastric cancer cells. Chem.-Biol. Interact..

[29] Chen D.Q., Han J., Liu H., Feng K., Li P. (2024). Targeting pyruvate kinase M2 for the treatment of kidney disease. Front. Pharmacol..

